# Cellular Tropism of SARS-CoV-2 across Human Tissues and Age-related Expression of ACE2 and TMPRSS2 in Immune-inflammatory Stromal Cells

**DOI:** 10.14336/AD.2021.0429

**Published:** 2021-06-01

**Authors:** Ming Zheng

**Affiliations:** ^1^Institute of Military Cognition and Brain Sciences, Academy of Military Medical Sciences, Beijing, China; ^2^Beijing Institute of Basic Medical Sciences, 27 Taiping Road, Beijing, China

**Keywords:** SARS-CoV-2, human tissues, ACE2, TMPRSS2, immune-inflammatory stromal cells

## Abstract

Recently, emerging evidence has indicated that COVID-19 represents a major threat to older populations, but the underlying mechanisms remain unclear. The pathogen causing COVID-19 is acute respiratory syndrome coronavirus 2 (SARS-CoV-2). SARS-CoV-2 infection depends on the key entry factors, angiotensin-converting enzyme 2 (ACE2) and transmembrane serine protease 2 (TMPRSS2). Recognizing the importance of ACE2 and TMPRSS2 for the cellular tropism of SARS-CoV-2, we analyzed and presented the landscape of cell-type identities for ACE2^+^ TMPRSS2^+^ cells across different human tissues and the age-related alterations in ACE2 and TMPRSS2 expression across different cell types. Additionally, most of the post-acute COVID-19 sequelae could attribute to the ACE2-expressing organ systems. Therefore, these SARS-CoV-2 tropism data should be an essential resource for guiding clinical treatment and pathological studies, which should draw attention toward the prioritization of COVID-19 research in the future. Notably, we discovered the age-related expression of ACE2 and TMPRSS2 in the immune-inflammatory stromal cells, implying the potential interplay between COVID-19, stromal cells, and aging. In this study, we developed a novel and practical analysis framework for mapping the cellular tropism of SARS-CoV-2. This approach was built to aid the identification of viral-specific cell types and age-related alterations of viral tropism, highlighting the power of single-cell RNA sequencing (scRNA-seq) to address viral pathogenesis systematically. With the rapid accumulation of scRNA-seq data and the continuously increasing insight into viral entry factors, we anticipate that this scRNA-seq-based approach will attract broader interest in the virus research communities.

As of 28 April 2021, the COVID-19 pandemic has resulted in 148,329,348 confirmed cases, including 3,128,962 deaths (https://covid19.who.int/). The pathogen causing COVID-19 is acute respiratory syndrome coronavirus 2 (SARS-CoV-2) [1]. SARS-CoV-2 infection begins when spike proteins attach to angiotensin-converting enzyme 2 (ACE2), the host cell receptor for SARS-CoV-2 [2]. Next, transmembrane serine protease 2 (TMPRSS2) cleaves the SARS-CoV-2 spike protein on the cell membrane, which is important for the fusion of viral and cellular membranes [3]. In summary, ACE2 is the prerequisite for SARS-CoV-2 infection, while TMPRSS2 facilitates viral entry and spread [3]. Therefore, the expression of ACE2 and TMPRSS2 directly points to the cellular tropism of SARS-CoV-2, which has important implications for the pathogenesis of COVID-19 [4, 5].

Recently, emerging evidence has indicated that COVID-19 represents a serious threat to older populations [6]. Older COVID-19 patients are disproportionately affected by severe and fatal outcomes. It is worth noting that 95.4% of COVID-19 death cases in the US are more than 50 years old [6]. Age-related alterations in gene expression play a pivotal role in human disease [7, 8]. Recognizing the importance of ACE2 and TMPRSS2 for the cellular tropism of SARS-CoV-2, we explored the landscape of cell-type identities for ACE2^+^ TMPRSS2^+^ cells across different human tissues and the age-related alterations in ACE2 and TMPRSS2 expression across different cell types.

A recent study identified various cell-type clusters in human tissues through single-cell RNA sequencing (scRNA-seq), reflecting the growing knowledge of cellular heterogeneity at the single-cell level [9]. Based on the well-established link between viral entry factors and cellular tropism [4, 5], we constructed a scRNA-seq-based analysis framework for mapping the cellular tropism by analyzing the expression of ACE2 and TMPRSS2. We consolidated the ACE2^+^ TMPRSS2^+^ cells by tissue type and cell-type cluster, and then established their relationships. Next, we evaluated age-related expression of ACE2 and TMPRSS2 in different cell types (Fig. 1). This approach was designed to enable a greater understanding of COVID-19 pathogenesis and to generate new hypotheses on which tissue and cell types are the potential targets of SARS-CoV-2.

**Figure 1.**
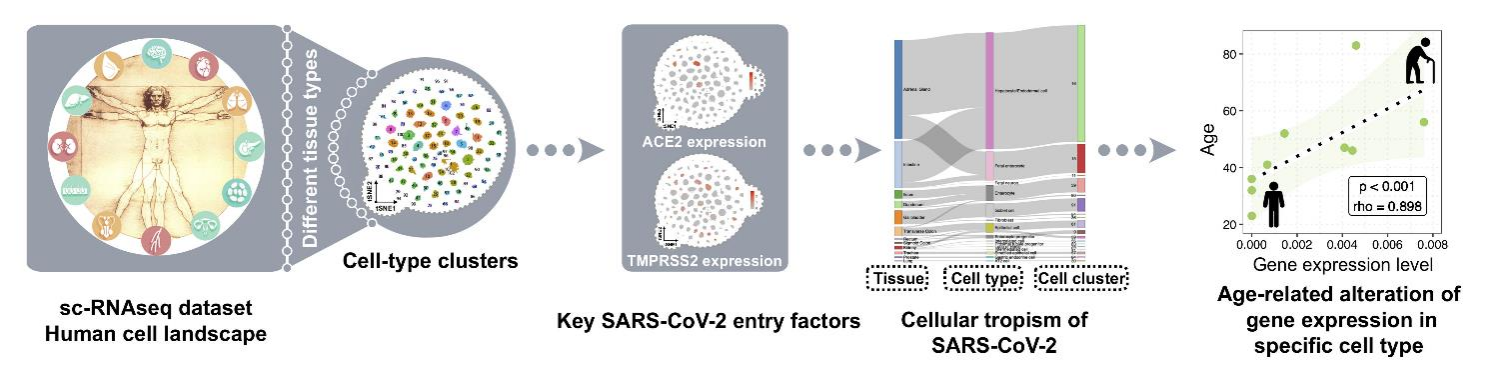
Graphical illustration of the analysis framework for mapping the cellular tropism of virus and age-related alterations. SARS-CoV-2-specific host cells were identified in different tissues, cell types, and cell clusters using the expression of the key SARS-CoV-2 entry factors, ACE2 and TMPRSS2. Age-related alterations in gene expression were analyzed in different cell types.

Through reanalyzing the scRNA-seq data of 599,926 single cells and 102 cell-type clusters from a previous study [9], we investigated the cellular tropism of SARS-CoV-2 at the single-cell level (Fig. 2A). We analyzed the scRNA-seq data using Seurat software [10]. The cell-type lineage annotations were retained from a previous study [9]. Next, we ranked the cell-type identities of ACE2^+^ cells according to the expression levels of ACE2 (Fig. 2B) and the percentages of ACE2^+^ cells (Fig. 2C). We found high ACE2 expression levels in hepatocyte/endodermal, epithelial, goblet, and proximal tubule progenitor cells. Next, we analyzed the relationship between the cell-type clusters and the tissue sources of ACE2^+^ cells (Fig. 2D). We discovered that individual ACE2-expressing cell-type clusters could be derived from various tissue types, revealing the complex relationship between the cell-type clusters and tissue sources for ACE2^+^ cells.

Next, we analyzed the overlapping expression of ACE2 and TMPRSS2 in cell and tissue types. Overlapping of ACE2 and TMPRSS2 expression levels were observed in heterogeneous cell types, including hepatocyte/endodermal, epithelial, goblet, proximal tubule progenitor, fetal enterocyte, and enterocyte cells (Fig. 2E-F), and various tissue types, including the intestine, duodenum, gallbladder, kidney, ileum, adrenal gland, and transverse colon (Fig. 2G-H). Next, we summarized the relationships between cell-type clusters and tissue sources for ACE2^+^ TMPRSS2^+^ cells (Fig. 2I).

Considering that the gene expression pattern might undergo substantial alterations during aging [7], we investigated the expression of ACE2 and TMPRSS2 in donors with age range of more than 50 years. The expression levels of ACE2 and TMPRSS2 in different cell types were measured by the average expression levels using more than 150 single cells; additionally, at least five donors were used for the Spearman correlation analysis [11], and *p*-values were corrected by the Bonferroni correction method [12]. Age-related alterations in ACE2 and TMPRSS2 expression were analyzed in different cell-type clusters (Fig. 2J-K). Notably, only cell cluster 27 (C27), annotated as stromal cells, showed age-related changes significantly. The ACE2 expression levels in C27 cells were significantly increased with age (*p* < 0.001, rho = 0.898; Fig. 2L), while the TMPRSS2 expression levels in C27 cells showed a slightly insignificant age-related correlation (*p* = 0.081, rho = 0.609, Fig. 2M).

**Figure 2.**
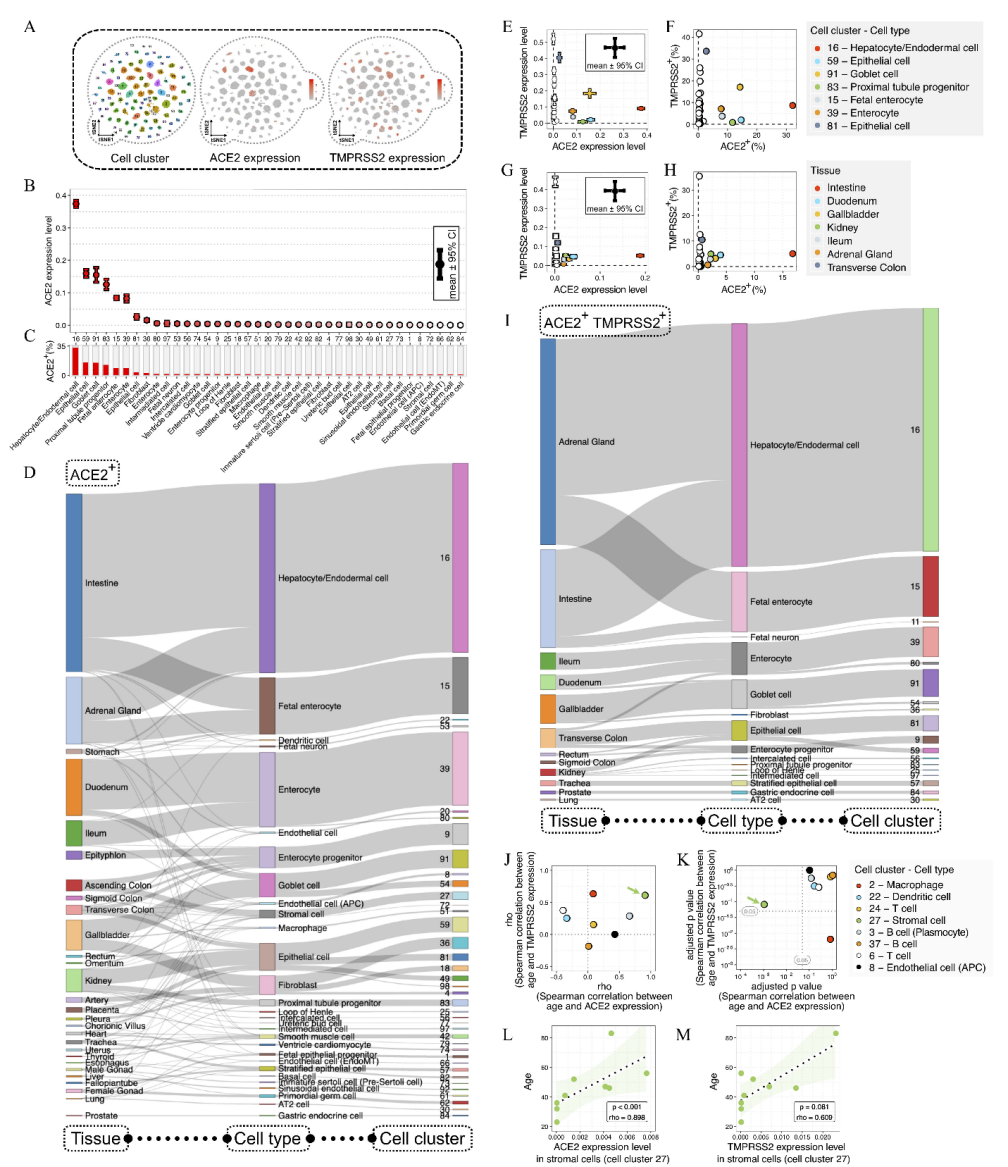
ACE2 and TMPRSS2 expression in different cell-type clusters across human tissues. (A) ACE2 and TMPRSS2 expression levels in different cell-type clusters. These cell types were clustered by single-cell transcriptome using the t-Distributed Stochastic Neighbor Embedding (t-SNE) method, with color coding according to cell-type clusters (left plot). The expression levels of ACE2 (middle plot) and TMPRSS2 (right plot) in different cell-type clusters. (B) Distribution of ACE2 expression levels across different cell-type clusters with more than 0.1% ACE2^+^ cells. The error bar represents the 95% confidence interval (CI) of ACE2 expression levels, with color coding according to the ACE2 expression levels. (C) Percentages of ACE2^+^ cells in different cell-type clusters. (D) Sankey plot shows the relationships among tissue sources, cell types, and cell clusters for ACE2^+^ cells. (E) The relationship between the expression levels of ACE2 and TMPRSS2 in different cell-type clusters. The error bars represent the 95% confidence interval (CI) of ACE2 and TMPRSS2 expression levels, with color coding according to cell-type clusters. (F) The relationship between the proportions of ACE2^+^ and TMPRSS2^+^ cells in different cell-type clusters. (G) The relationship between the expression levels of ACE2 and TMPRSS2 in different tissues. The error bars represent the 95% confidence interval (CI) of ACE2 and TMPRSS2 expression levels, with color coding according to tissue types. (H) The relationship between the proportions of ACE2^+^ and TMPRSS2^+^ cells in different tissues. (I) Sankey plot shows the relationships of tissue sources, cell types, and cell clusters for ACE2^+^ TMPRSS2^+^ cells. (J-K) The correlations between age and the expression levels of ACE2 and TMPRSS2 in different cell-type clusters. Spearman's rho coefficients (J) and Bonferroni-adjusted *p*-values (K) for the correlations in different cell-type clusters. (L-M) The correlations between age and the expression levels of ACE2 (L) and TMPRSS2 (M) in stromal cells (cell cluster 27).

**Figure 3.**
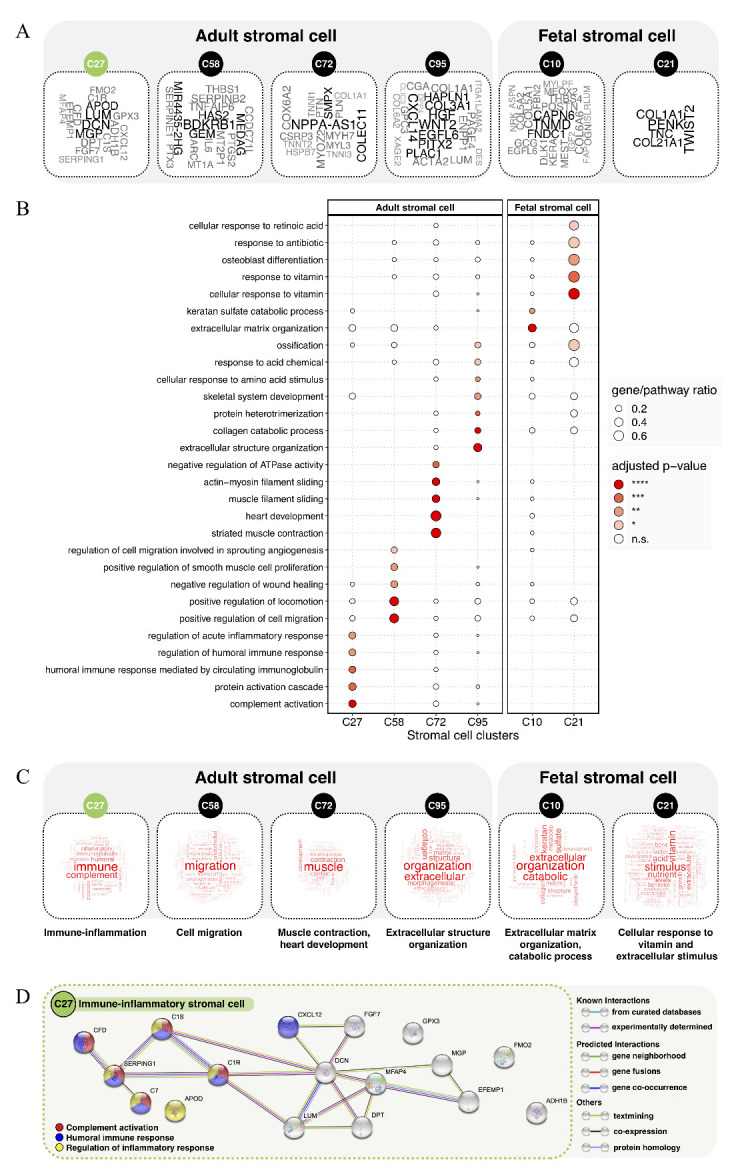
Characterizing the phenotypes of different stromal cell clusters. (A) Word cloud plots show the feature genes of different stromal cell clusters, including adult stromal cells (C27, C58, C72, C95) and fetal stromal cells (C10, C21). The feature genes were selected from the differentially expressed genes in each cell cluster against all the other cell clusters (log fold-change > 4, Bonferroni-adjusted *p*-value < 0.0001, expressed in > 15% of cells in either cell-type cluster). The word size reflects the fold-change of gene expression. (B) GO pathway enrichment analysis of feature genes in different stromal cell clusters. Dot plot shows the most significantly enriched GO terms of biological processes (Bonferroni-adjusted *p*-values < 0.05). The dot size shows the gene-pathway ratio, and the dot color shows the pathway enrichment significance. (C) Word cloud plots show the feature pathways in different stromal cell clusters. The word size reflects the pathway enrichment significance. (D) The STRING interaction network for immune-inflammatory stromal cells (C27). The interaction network uses the feature genes of C27, with node color coding according to the significantly involved immune-inflammatory pathways (Bonferroni-adjusted *p*-values < 0.05), including complement activation (red), humoral immune response (blue), and regulation of inflammatory response (yellow).

It is worth noting that C27 is one of the six stromal cell clusters, which include adult stromal cells (C27, C58, C72, C95) and fetal stromal cells (C10, C21) [9]. Stromal cells are very heterogeneous cell populations, performing diverse biological functions [13]. Thus, our knowledge is far from complete, especially regarding the functional role of different stromal cell clusters. Based on the premise that biological functions are controlled by gene expression, we aimed to elucidate the key biological functions by analyzing the feature genes of different stromal cell clusters. The feature genes were selected from differentially expressed genes in each cell cluster against all the other cell clusters (Fig. 3A). Next, the feature genes were used for GO pathway analysis, as previously reported [14]. The results of pathway analysis (Fig. 3B) are presented as word cloud visualizations (Fig. 3C), indicating the following biological functions: immune-inflammation (C27); cell migration (C58); muscle contraction, heart development (C72); extracellular structure organization (C95); extracellular matrix organization, catabolic process (C10); and cellular response to vitamin and external stimulus (C21).

To extend the above finding, the feature genes of C27, including DCN, LUM, MGP, APOD, DPT, ADH1B, CFD, C7, C1S, C1R, FGF7, GPX3, EFEMP1, CXCL12, FMO2, SERPING1, and MFAP4, were mapped in the STRING interaction network [15]. We found that 41.1% of the C27 feature genes were significantly enriched in the key pathways of immune-inflammation, including complement activation, humoral immune response, and regulation of inflammatory response (Bonferroni-adjusted *p*-values < 0.05). Moreover, for the immune-inflammatory function of C27, interactions between C1S, C1R, SERPING1, CFD, and C7 were actively involved (Fig. 3D).

Currently, emerging evidence has indicated that multiple tissues are vulnerable to COVID-19 [16]. In this study, we discovered that the digestive system (e.g., intestine, duodenum, gallbladder, ileum, and transverse colon) and kidney have significantly higher expression levels of ACE2 and TMPRSS2. Given the critical role of ACE2 and TMPRSS2 in SARS-CoV-2 infection [3], the higher expression of ACE2 and TMPRSS2 may contribute to the higher vulnerability to COVID-19 in these tissues. These findings are consistent with the clinical manifestations of COVID-19. Several related COVID-19 symptoms, including kidney failure and gastrointestinal symptoms (e.g., diarrhea), have been reported [17, 18]. Moreover, detectable viral RNA of SARS-CoV-2 has been found in the fecal samples from COVID-19 patients [19]. Thus, it is almost certain that the digestive system should be involved in SARS-CoV-2 infection. In addition, we found that the adrenal gland might also be highly vulnerable to SARS-CoV-2, implying a potential relationship between the COVID-19 and the endocrine system. Altogether, we dissected the tissue types that appear to be highly permissive to SARS-CoV-2, which likely lead to the complicated clinical manifestations and outcomes. Further research should pay close attention to multiple tissues attacked by SARS-CoV-2.

Beyond the acute COVID-19 phase, some COVID-19 survivors have a higher risk of death and suffer persistent long-lasting post-acute sequelae of COVID-19 [20, 21]. The post-acute sequelae of COVID-19 include both pulmonary and extrapulmonary manifestations, spanning a wide breadth of organ systems [21]. Here, we leveraged the post-acute COVID-19 sequelae data [21] and compared the relationships between the COVID-19 sequelae in multiple organ systems and the status of ACE2 and TMPRSS2 expression. We found most of the post-acute COVID-19 sequelae could attribute to the ACE2-expressing organ systems. Moreover, several involved organ systems exhibited the expression of both ACE2 and TMPRSS2, which include endocrine, gastrointestinal, kidney, and pulmonary tissues (Fig. 4). This finding indicates that the post-acute sequelae of COVID-19 might be caused by SARS-CoV-2 infection directly.

Gastrointestinal disorders and kidney diseases have been reported in both acute and post-acute phases of COVID-19 [17, 18, 21]. Consistently, the SARS-CoV-2-permissive ACE2^+^ TMPRSS2^+^ cells could be found in the gastrointestinal system (including multiple organs of intestine, duodenum, gallbladder, ileum, and transverse colon) and kidney (including multiple cell-type clusters of epithelial, intercalated, proximal tubule progenitor, loop of henle, and intermediated cells). Although the tissue locations of ACE2^+^ TMPRSS2^+^ cells might partly explain the signs and symptoms of COVID-19, other indirect conditions might also be involved, which include social, economic, and behavioral differences. Beyond these indirect conditions, a deeper understanding of the underlying mechanisms is still required for SARS-CoV-2 infection.

A previous study successfully isolated SARS-CoV-2 from human airway epithelial cells [1]. Here, we found that different epithelial cell clusters, including C59, C81, C38, C49, C57, C60, C82, and C98, might have different degrees of predisposition to SARS-CoV-2. Among all the 102 cell clusters, C59 cells had the second highest ACE2 expression level but a relatively low TMPRSS2 expression level. In contrast, C81 cells had the highest TMPRSS2 expression level but a relatively low ACE2 expression level. Comparatively, other epithelial cell clusters had relatively low or undetectable expression levels of ACE2 and TMPRSS2. Moreover, it is worth noting that several cell-type clusters had remarkably higher expression levels of ACE2 and TMPRSS2 than epithelial cells. For example, C16 cells (hepatocyte/endodermal cells) had the highest ACE2 expression level and a moderate TMPRSS2 expression level. Thus, our knowledge of SARS-CoV-2 infection is far from complete. These heterogeneous ACE2^+^ TMPRSS2^+^ cells might represent the missing pieces of the jigsaw puzzle, and further studies are required to justify these findings.

**Figure 4.**
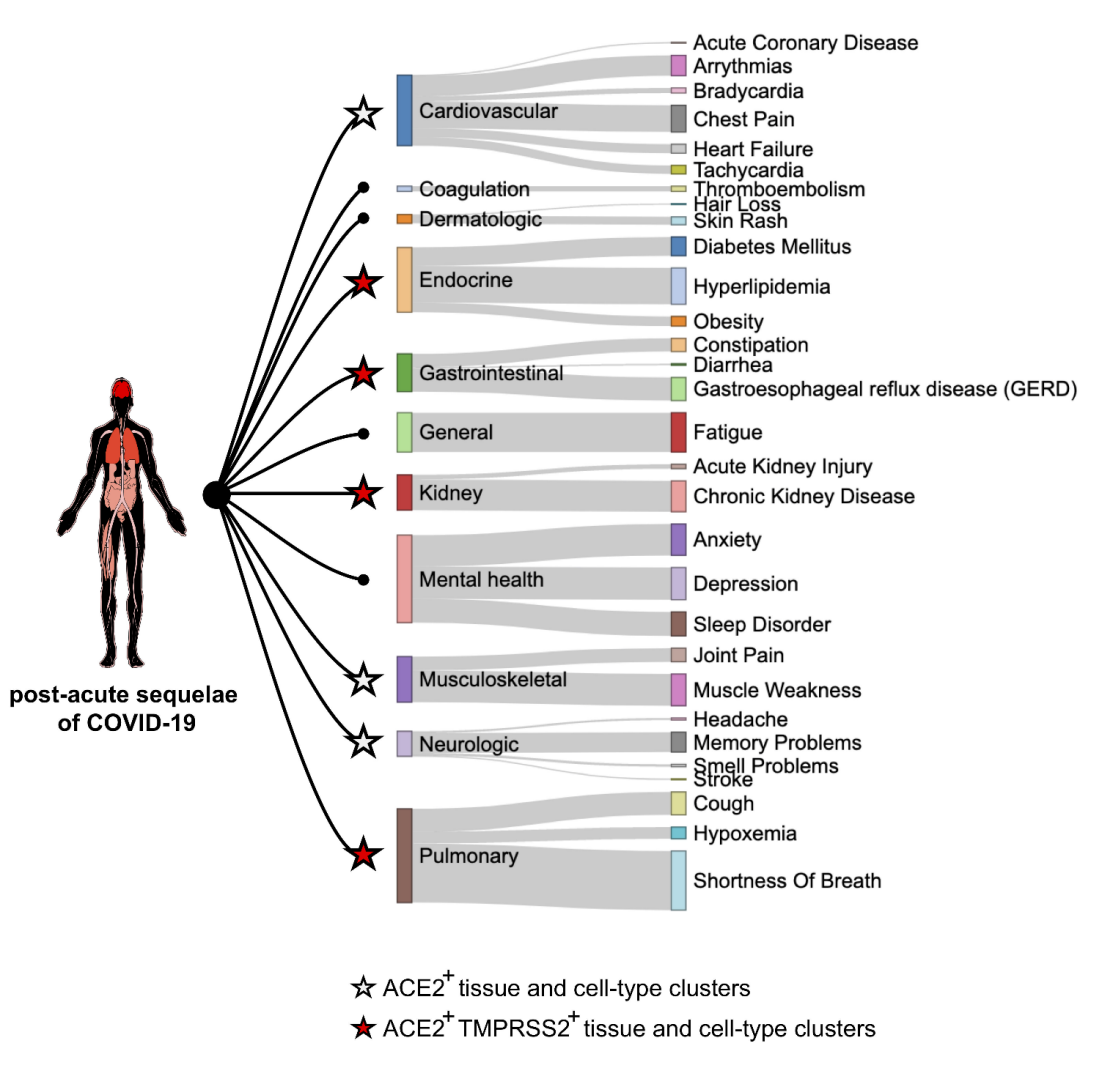
Post-acute COVID-19 sequelae in multiple organ systems and the expression of ACE2 and TMPRSS2. Human body plot illustrates organ systems affected by post-acute COVID-19 sequelae, with color coding according to the excess burdens of sequelae. Sankey plot shows the relationships among post-acute COVID-19 sequelae, involved organ systems, and expression of ACE2 and TMPRSS2. Node size shows the excess burden of clinical outcome per 1000 participants at 6-months in COVID-19 cases compared to the US VHA (Veteran Health Administration) users. Star shape and color show the status of ACE2 and TMPRSS2 expression in related tissues and cell-type clusters.

It has been reported that older populations, especially those 50 years old or older, are more vulnerable to COVID-19 [6]. Among various ACE2^+^ TMPRSS2^+^ cell clusters, only the C27 stromal cells showed a noteworthy increased expression of ACE2 and TMPRSS2 in older people, especially those aged more than 40 years old. Furthermore, our analyses also reasonably depicted a preliminary portrait of C27, the immune-inflammatory stromal cells. The disease severity of COVID-19 is due to not only SARS-CoV-2 infection but also the immune-inflammatory response [22]. The uncontrolled immune-inflammatory response causes multi-organ damage and failure, leading to cytokine storm in fatal COVID-19 cases [23]. Collectively, our findings might depict the complex interplay between SARS-CoV-2, stromal cells, immune-inflammation, and aging.

The most well-acknowledged role of stromal cells is to provide tissue repair and renewal following aging and illness [24]. Previous studies have successfully used stromal cells to treat active diseases [13], including COVID-19 [25]. Recently, the therapeutic usage of mesenchymal stromal (stem) cells (MSCs) has been undergoing clinical trials in COVID-19 patients [26]. For such clinical trials, it should be important to consider the inclusion of older participants, analyses stratified by age range, and enrichment of ACE2^-^ cells for therapeutic usage.

Due to the low sequencing coverage of scRNA-seq data, our analysis might have the limitations of not fully covering those cells with relatively low ACE2 and TMPRSS2 expression. Some recent scRNA-seq-based approaches, such as targeted and oligonucleotide-labeled antibody sequencing, should be promising techniques for solving this problem [27, 28]. Although our analysis reveals a considerable number of ACE2^+^ TMPRSS2^+^ cell types, further efforts using alternative technologies should be required to achieve a more comprehensive picture of ACE2^+^ TMPRSS2^+^ human cells. Additionally, although the variation in ACE2 and TMPRSS2 expression may partially contribute to COVID-19 risk, many other risk factors for COVID-19 should also be considered, such as pre-existing medical conditions of cardiovascular diseases, diabetes, and chronic respiratory diseases [29].

Despite the growing body of evidence suggesting the multi-organs and multi-cell types infected by SARS-CoV-2 in previous and current studies, we still lack well-founded experimental results that explain how viral tropism can shape COVID-19 pathogenesis. Nevertheless, these SARS-CoV-2 tropism data should be an essential resource for guiding clinical treatment and pathological studies, which should draw attention toward the prioritization of COVID-19 research in the future.

Moreover, we developed a novel and practical analysis framework for mapping the cellular tropism of SARS-CoV-2. This approach was built to aid the identification of viral-specific cell types and age-related alterations of viral tropism, highlighting the power of scRNA-seq to address viral pathogenesis systematically. With the rapid accumulation of scRNA-seq data and the continuously increasing insight into viral entry factors, we anticipate that this scRNA-seq-based approach will attract broader interest in the virus research communities.
